# Effects of Vocational Re-training on Employment Outcomes Among Persons with Disabilities in Germany: A Quasi-Experiment

**DOI:** 10.1007/s10926-019-09866-x

**Published:** 2019-11-28

**Authors:** Nicolas Echarti, Esther Schüring, Cathal O’Donoghue

**Affiliations:** 1 Maastricht Graduate School of Governance, Maastricht University, Maastricht, The Netherlands; 2 University of Applied Sciences Bonn-Rhein-Sieg, Sankt Augustin, Germany; 3 National University of Ireland, Galway, Ireland

**Keywords:** Return to work, Rehabilitation, Vocational re-training, Program effectiveness, Propensity score

## Abstract

*Purpose* To investigate how completing vocational re-training influenced income and employment days of working-age people with disabilities in the first 8 years after program admission. The investigation also included the influence of vocational re-training on the likelihood of receiving an earnings incapacity pension and on social security benefit receipt. *Methods* This retrospective cohort study with 8 years follow up was based on data from 2399 individuals who had completed either a 1-year vocational re-training program (n = 278), or a 2-year vocational re-training program (n = 1754) or who were admitted into re-training but never completed the program (n = 367). A propensity score-based method was used to account for observed differences and establish comparability between program graduates and program dropouts. Changes in outcomes were examined using the inverse probability-weighted regression adjustment method. *Results* After controlling for other factors, over the 8 years after program admission, graduates of 1-year re-training, on average, were employed for an additional 405 days, 95% CI [249 days, 561 days], and had earned €24,260 more than without completed re-training, 95% CI [€12,805, €35,715]. Two-year program completers, on average, were employed for 441 additional days, 95% CI [349 days, 534 days], and had earned €35,972 more than without completed re-training, 95% CI [€27,743, €44,202]. The programs also significantly reduced the number of days on social-security and unemployment benefits and lowered the likelihood of an earnings incapacity pension. *Conclusion* Policies to promote the labor market re-integration of persons with disabilities should consider that vocational re-training may be an effective tool for sustainably improving work participation outcomes.

## Introduction

Although it has been shown that there are many benefits to hiring people with disabilities [1], people with a disability still face considerable economic disadvantages compared to working-age people without disabilities. Disadvantages include lower employment rates and a significantly higher risk of living in poverty [2, 3]. Other consequences of prolonged unemployment include a lower quality of life and reduced social inclusion. The situation also constitutes a major public concern since low employment rates among people with disabilities are in many ways a challenge to economic productivity and the financial stability of social security systems [4].

These factors have led to numerous occupational rehabilitation studies, some of them examining the effectiveness of interventions promoting re-employment. However, whereas systematic evidence shows how interventions implemented at the workplace impact employment outcomes [5–12], there is less conclusive evidence on the effects of (out-of-job) re-training measures. Some empirical studies indicate a positive effect on income and employment [13–17] while other authors find little or no effects resulting from program participation [18, 19]. Interpreting these findings is complicated by differences in study populations, methods used and in the vocational education measures analysed, which may have different mandates, strategies and curricula.

A common problem throughout the analysis of vocational re-training measures is that researchers generally struggle to recruit study participants, which may limit the types and appropriateness of methods being used [20]. Due to their individual health situation, people with a disability are a heterogeneous group, making it difficult to find suitable comparison groups for program evaluation [21]. Individuals who are deemed eligible to participate in rehabilitation measures usually cannot be denied access to the services. The challenge in evaluating the impact of an intervention is thus to obtain a credible estimate on the counterfactual: What would have happened to the graduates of the re-training programs had they not completed the measures?

In the absence of a natural comparison group, an alternative is to draw a comparison with program applicants who were admitted into a re-training program but never received the full benefit from training (due to no-show or dropout). Drawing comparison with an applicant-based comparison group has several advantages compared to impact analysis based on an external comparison group design. While program graduates and dropouts share the same motivation to apply for vocational education, satisfy the eligibility criteria, and potentially have similar health problems, selection bias is minimized [22]. Moreover, if data is process generated, distortions due to response denials or omissions in the retrospective collection of data can be dismissed.

Using data from Germany as a case study, we utilized rich administrative data of a cohort of re-training applicants to provide new and unique evidence on the effects of vocational re-training on long-term employment outcomes. By drawing a comparison with a group of similar program non-completers, we add to a continuing methodological debate on how to estimate program effects from observational data. A successful outcome to this debate could improve public policy making. While adjusting the results for measured confounders, we show to what extent completion of a re-training program influenced subsequent income and employment development. Additionally, we evaluated the influence of the re-training programs on the number of days with unemployment and social-security benefits and with regards to the uptake of a pension due to a limited earnings capacity.

The remainder of this article is structured as follows: In the next section, the methods are presented, with a description of the study design, the study population, the re-training programs and the data used. Moreover, the method of statistical analysis is summarized. In the results section, descriptive statistics of the participants are presented and the balance in covariates between comparison groups is evaluated, before treatment effects of the re-training measures are presented. In the final section of this article, we discuss the implications and shortcomings of the findings before offering our conclusions.

## Methods

### Study Design

To investigate the employment effects associated with vocational re-training in Germany, a retrospective, quasi-experimental, cohort study was performed. In the absence of a natural control group, an internal comparison group design was used to compare outcomes with and without completed vocational re-training. The impact of the re-training measures was assessed by estimating average treatment effects of graduating from a re-training program in comparison to the scenario of unsuccessful program completion (program dropout). The data used for this analysis was collected from the administrative data records of the *German Statutory Pension Insurance Fund* (*Deutsche Rentenversicherung Bund).* They featured accurate, process-generated information on the earnings development and insurance relationship for a cohort of rehabilitants in the 8 years after admission into re-training and 2 years prior.

### Study Population and Eligibility Criteria

The study population consisted of people with disabilities who, due to their health problem, were no longer able to (or were it was predicted that they will in the foreseeable future no longer be able to) carry out prior job tasks. Additionally, they must have successfully applied for vocational rehabilitation with the *German Statutory Pension Insurance Fund*. To become eligible for vocational re-training with the *German Pension Insurance*, candidates must satisfy any of the following eligibility criteria: Either the applicants (1) already receive a pension due to limited earnings capacity, or (2) an assessment has been made that, without the measure, the pension provider would have to pay out a pension due to limited earnings capacity of the person with a disability, or (3) a medical rehabilitation alone was determined to be insufficient for proper reintegration of a person with a disability into the labor market, or (4) the 15-year waiting period has been completed. Additionally, the training scheme needs to be considered necessary for the rehabilitant and there should be a positive chance of a successful measure and consequent re-employment. This success is, for instance, likely if the chances of employment in the rehabilitant’s target occupation are good but require additional skills or job specific knowledge. Once the application for rehabilitation has been approved, suitable re-training programs are chosen from a large pool of certified private or public institutions specialized in different professions and skill training.

### Interventions

Vocational re-training in Germany can generally be categorized into partial and full vocational re-training programs. Apart from the fact that both types of re-training programs require full-time participation, they differ considerably in contents and length: Full re-training programs are comparable to regular apprenticeships programs, typically lasting for 2 years. During the programs, class-room training is combined with on-the-job training to learn skills and obtain a professional qualification in a new field of work. A formal examination is often set at the end of the programs. Partial qualification measures, on the other hand, aim at extending existing competences with additional skills, e.g. in the fields of business administration or information technology. The measures are usually completed within a year, aiming to reintegrate the participants more quickly into working life. In the light of political initiatives to strengthen horizontal training (qualification for additional tasks), partial qualifications are becoming increasingly popular as an alternative to the more involved full re-training programs.

### Data Collection

The data used for this analysis was retrieved from administrative records made available by the *Research Data Centre of the German Statutory Pension Insurance Fund.* The corresponding data (SUF_RSDV2013) is representative of the whole rehabilitant population and featured accurate, process-generated information on the income and employment development before and after participation in a rehabilitation measure. Additionally, it was also possible to link the data to personal socio-economic and health information. The retrieved data consisted of four databases: The first database included a sample of all vocational re-training cases in the year 2005 and a range of variables linked to implementing the rehabilitation programs. Moreover, it provided information on some labor-market-related and personal characteristics at the time of application for rehabilitation. The data included information on the type of granted rehabilitation measure, the rehabilitation start date, the medical discharge diagnosis (ICD), the employment status and the residential region at the time of application as well as an indicator variable reflecting whether the rehabilitant has successfully completed the re-training measures. The second dataset was retrieved from the pension insurance follow-up database, providing information on the participants’ insurance relationships. It consisted of observations from 2003 to 2013, including annual individual income and days of (un-) employment data, overcoming the lack of detailed labor market data present in many other studies. In this study, the following variables were used from this file: year, yearly income, annual days of employment, annual days with short- and long-term unemployment, annual days with other social-security benefits and the occupational group in which the individuals was working prior to program start. To provide further information on the participants’ socio-demographics, a third dataset was merged with the previous files. The latter file included data on the persons’ sex, birth and death years, nationalities and highest attained levels of education. Lastly, a fourth dataset provided information on receiving a pension. The data comprised information on whether a pension due to a reduced earnings capacity was paid.

### Comparison Groups

In order to compare the impact of the re-training programs a trivariate indicator variable was created, reflecting whether the rehabilitant has either dropped out of re-training (did not successfully complete the measures; value = 0), successfully completed a 1-year re-training (value = 1) or successfully completed a 2-year re-training (value = 2). The outcome of the vocational re-training measure was identified using the variable “BFEWMS” from the provided dataset.

### Outcome Measures

Participation in paid employment was assessed using nominal and inflation-adjusted income, as well as employment days, in the first 8 years after program admission (2006–2013). This data was collected directly from the provided pension insurance follow-up database. A potential drawback of the data used was that employment data collected by the German pension insurance was limited to a contribution ceiling. For annual incomes above €69,600 (€58,800 in the former East German states), the median value of €77,179 (€65,400) was recorded in the data instead of the true value. This potentially lowered the estimated program impact, as annual income above the specified cut-off value were not properly recorded. Changes in real income were estimated using the average of historical inflation rates collected from the *German Federal Bureau of Statistics* between 2003 and 2013. The mean annual discount factor used was equal to 1.6%.

Secondary outcomes comprised days on social-security benefits, days on short-term unemployment benefits, days on long-term unemployment benefits and an indicator variable signaling whether a pension due to a reduced earnings capacity was awarded. The data on social-security benefits included sick pay and temporary allowance payments made the during period of rehabilitation. Short-term unemployment benefits are benefits of the German unemployment insurance, which are paid on the occurrence of unemployment. They are usually paid for up to 1 year, and for older unemployed people, for up to 2 years. Long-term unemployment benefits are the basic (means tested) social security benefits for employable persons in Germany, who are unemployed for longer than 12 months. Data on days with long-term unemployment benefits was only available from 2006 to 2009. Workers who are only able to work a few hours a day because of their health can apply for a reduced earnings capacity pension. An individual can claim a reduced earnings capacity pension, if for the foreseeable future, because of ill health or disability, they are unable to do more than 3 h of paid work a day.

### Independent Variables

The following variables were included as independent variables in the calculations: We collected the age, gender and level of education from the provided demographic database. Additionally, the residential region, the main medical diagnosis and the employment status were retrieved from data collected during the application process. Moreover, based on the information provided from the insurance follow-up database, we included several measures of past earnings performance. These were the nominal income earned in the year 2003 and 2004 as well as the last registered occupation type.

### Econometric Approach

Adopting the counterfactual framework pioneered by Rubin [23], we tested whether completing re-training significantly improved the employment situation compared to the case of not completing re-training. In this framework a causal effect can be inferred from the difference between two potential outcomes; one that occurs if a person completes a re-training measure, and one that occurs if they do not complete re-training. In our analysis, we made use of pre-treatment micro data on the rehabilitants’ socio-economic status to estimate conditional treatment probabilities (propensity scores) in order to re-weight observations and balance the measured covariates across comparison groups. Through re-weighting observations, by the inverse probability of being treated, a comparison group with the same distribution of observables as in the treatment group is implicitly created [24]. Because there are a variety of observable factors that can be linked to return-to-work outcomes, it can be cumbersome to determine along which dimension to compare treated and non-treated subjects. Propensity scores, on the other hand, provide a natural weighting scheme that allows the observed differences between comparison groups to be minimized. To examine changes in employment outcomes, the average treatment effect (ATE) and the average treatment effect on the treated (ATET) were consequently estimated using the inverse probability weighted regression adjustment method (IPWRA).

### Propensity Score Model

The developed specification of the propensity score in this analysis included a variety of different variables thought to be characterizing the earnings ability of the rehabilitants. The factors used to predict program allocation/completion can be grouped into individual characteristics, health information, and economic variables on pre-treatment employment outcomes. There is no comprehensive list of variables that would make sure that the re-weighting procedure can provide an unbiased estimate of the program effect. However, knowledge of the institutional criteria that govern program allocation and the factors that have been found to be strongly correlated to subsequent labor market outcomes, are a good starting point.

One is not guided by the statistical power or significance of the estimated regression coefficients in the selection stage. Rather the objective is to create a sample in which the distribution of covariates that affect labor market outcomes between treated and control subjects is similar, and, thereby, include enough variables for the (weak form of) the conditional independence assumption to hold. This is achieved when adding additional variables that do not (significantly) alter the estimated coefficients anymore.

Using Statas user-written *“bfit”* command, combinations of explanatory variables can be tested to identify models that exhibit high efficiency based on the combination of covariates included. The statistical criteria tested to establish efficiency were the Bayesian Information Criterion (BIC) and the Akaike Information Criterion (AIC). Both, the BIC and the AIC, are Penalized-likelihood information criteria that enable cross-comparison of different models using statistical power analysis [25]. The two information criteria support different models depending on the trade-off between the relative importance one assigns to specificity versus sensitivity.

Using all variables specified in the data section and allowing second order polynomials as well as interaction terms, all possible combinations of covariates were tested regarding their joint significant and variation explained in relation to the main outcome variable; i.e. the sum of income earned in the years 2006–2013. In accordance with theory, in our estimations, the model with the lowest BIC had a higher joint significance compared to the model with the lowest AIC, an F-statistic of 44.36 vs. 28.91, whereas the model with the lowest AIC was able to explain more of the variation in the dependent variable (Adj. R-squared = 0.2391 vs. 0.2244).

The final specification used in our analysis included all variables suggested by the model with the lowest BIC. Additionally, three further groups of variables were added to the model as suggested by the AIC in order to increase the amount of variation explained. These were an interaction term between before application earnings from 2004 and age at admission, indicator variables reflecting the highest attained level of education and indicator variables for the registered medical diagnosis. Three other terms (Wage2004^2^, Age^2^ and Wage2003*Age), included in the model with the lowest AIC, were not used in the final specification because they correlated strongly with already included variables, while not being existential for the (weak form of the) conditional independence assumption to hold (they did not significantly alter the estimated coefficients when included). Some of the variables, in the final regression model were not individually statistically significant but the likelihood ratio test statistic for the model was large (Prob > χ^2^ = 0.000). The F-statistic and adjusted variation explained of the final model were in between the models with the optimal BIC and AIC (F-statistic=33.80; Adj. R-squared = 0.2366).

The final specification of the propensity score model to estimate the conditional treatment (graduation) probabilities took the following functional form:

$$\begin{aligned} &{\text{Mlogit}}\;\left( {{\text{treatment}}} = 0,\;1,\;2 \right) \\ &\quad = \beta_{0} + \beta_{1} \times {\text{Wage}}\; 2003^{2} + \beta_{2} \times {\text{Wage }}\;2003 + \beta_{3} \times {\text{Wage }}\;2004 + \beta_{4} \times {\text{Wage}} \;2004 \\ &\quad\quad \times {\text{Age at admission}} + \beta_{5} \times {\text{Age at admission}} + \beta_{6} \times {\text{Working Status at admission}} \\ & \quad\quad + \beta_{7,8,9 \ldots 15} \times {\text{Job Type at admission}} + \beta_{16} \times Sex + \beta_{17 - 20} \times {\text{Education level}} + \beta_{21,22} \\ & \quad\quad \times {\text{ICD diagnosis}} + \beta_{23} \times {\text{Region}} + e \end{aligned}$$

### Outcome Model

The IPWRA estimator makes use of conditional probability weights to obtain outcome-regression parameters that account for the missing-data problem, which arises from each subject being observed in only one of the potential outcomes. The IPWRA method is characterized by a three-step procedure to estimate treatment effects:

1. The propensity score was estimated using multinomial logistic regression in order to establish comparability, modelling the program completion status as the dependent variable and individual characteristics as independent variables. Our analysis made use of Imbens’ generalization of the propensity score [26], which showed that the results of Rosenbaum and Rubin [27] continue to hold when the treatment is multi-valued. Using Statas’^1^ built-in “*mlogit*” command, conditional treatment probabilities were estimated. The “*mlogit*” command fits maximum-likelihood multinomial logit models, also known as polytomous logistic regression. In line with Wooldridge’s advice [28], we included into our specification all covariates that were correlated with employment outcomes, even though they were not individually significant in the selection model. Following Rosenbaum and Rubin’s approach [29], an iterative approach to specifying the propensity score model was used; quadratic- and interaction-terms were added accordingly (shown in the previous section).
2. Regression models of the employment and social security outcomes were fitted for each treatment level separately (OLS for continuous outcomes, Probit for discrete).^2^ In the regression models, observations were weighted by the inverse probability of treatment (i.e. the conditional probability of people participating in a program). The results of the models were used to predict treatment specific potential outcomes for each subject in both the treated and the non-treated state (of which only one is observed in reality).
3. The means of the treatment-specific outcomes were computed. The differences in these averages provided the estimates of the average treatment effects (ATE). In addition, because researchers and policy makers might be more interested in the causal treatment effects only for those who successfully completed the re-training measures, the average treatment effects on the treated (ATET) were calculated. This was achieved by limiting the observations to the subset of graduates before estimating the mean differences between the outcomes with and without program completion.

Following these steps produces consistent estimates of the influence of the intervention because the treatment is assumed to be independent of the potential outcomes after conditioning on the covariates [30]. Hereby, it is assumed that all differences between program graduates and dropouts are due to observable characteristics. The ipwra estimator exhibits the double robust property meaning that it applies the selection and outcome model simultaneously, thus, producing a consistent estimate of the parameters if either of the two models is correctly specified [28].

Post-estimation statistics were, henceforth, computed to describe the extent to which covariates were balanced across comparison groups after re-weighting of observations. In particular, Let ${{\bar{x}}_{gj}}$ refer to the sample average of covariate *j* for the treatment groups *g *= 0,1,2 and let ${{s}_{gj}}$ be the sample standard deviation; the calculation of normalized differences in a variable then takes the following form [31]:

$${\text{Standardized difference in continuous variable}} = \frac{{\left( {\bar{x}_{1j} - \bar{x}_{0j} } \right)}}{{\sqrt {\frac{{\left( {s^{2}_{1j} + s^{2}_{0j} } \right)}}{2}} }}$$

If the variable is measured on a discrete scale, the standardized differences were calculated as follows:

$${\text{Standardized }} {\text{ difference }} {\text{ in }} {\text{ discrete }} {\text{ variable}} = \frac{{\left( {\hat{p}_{1j} - \hat{p}_{0j} } \right)}}{{\sqrt {\frac{{\hat{p}_{1j} \left( {1 - \hat{p}_{1j} } \right) + \hat{p}_{0j} \left( {1 - \hat{p}_{0j} } \right)}}{2}} }}$$

where $\widehat{{{p}_{1j}}}$ and $\widehat{{{p}_{0j}}}$ denote the proportion or mean of a binary baseline variable in the treatment and control group, respectively. Although there is no clear cut-off point defined, 0.10 and 0.25 have frequently been considered as values the estimates should be compared against. In general, a difference in average means larger than 0.25 standard deviations are substantial, and, in that case, suspicion may be called for [32]. In contrast, standardized differences as low as 0.10 reflect a degree of balance comparable to what one might expect in a completely randomized experiment [31].

## Results

### Participants

**Fig. 1:**
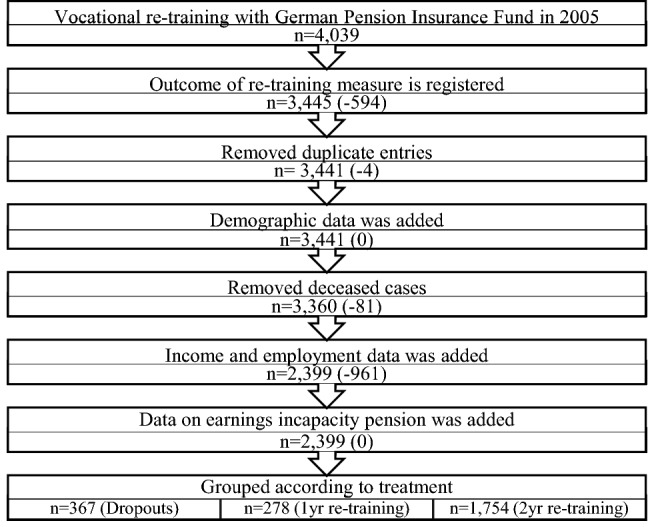
Sampling process

Figure 1 reports the numbers of individuals at each stage of the study. The unique identifier, present in all four databases used to merge the data, was the variable “case”. Overall, there were 4039 observations in the initial sample with program start in 2005. Limiting the observations to those, for which the program outcome has been registered, reduced the sample to 3445 observations. Furthermore, duplicates were removed, which reduced the sample by four observations. Next, demographic data was added for all remaining observations. Given the information retrieved in the demographic data file, persons that passed away during the observation period were dropped from the sample. This further reduced sample size to 3360 observations. To retrieve information on the employment status before and after participation in vocational rehabilitation longitudinal labor market data was then merged with the rehabilitation data. Due to interrupted or missing income and employment records, 961 observations were dropped from the sample. In a last step, data on pension receipt was added for those cases, in which a pension due to a reduced earnings capacity was awarded.

The final sample consists of 2399 individuals with complete employment records from 2003 to 2013. In total, the group of program dropouts contained 367 observations, while there were 278 cases with successfully completed 1-year re-training and 1754 cases with successfully completed 2-year re-training. The group of program dropouts included both 1-year and 2-year program dropouts due to the rather small number of controls available. Out of the 367 observations that started any of the re-training programs, but never completed, 328 persons started a 2-year re-training, while only 39 persons started a 1-year program.

In Table 1 the registered reasons for program dropout are listed to provide background information on the causes of unsuccessful program participation. Medical reasons were the predominant reason for program dropout followed by failed examination and other performance issues. Whether this had an influence on the estimated relationships was tested in a sensitivity analysis, added to the end of the results section, which excluded program dropout cases with a registered medical reason for program dropout, from the impact analysis. For the main part of the analysis, all program dropouts (n = 367) were used as controls.

**Table 1:** Registered reason for program dropout

Dropout reason	Freq.	Percent
Medical (patient)	177	48.23
Failed examination	80	21.80
Performance (insurer)	24	6.54
Other (insurer)	23	6.27
Other (patient)	20	5.45
Performance (patient)	16	4.36
Personal (patient)	15	4.09
Disciplinary (insurer)	9	2.45
Economic (patient)	3	0.82

### Descriptive Characteristics of Study Participants

Table 2 contains an overview of the descriptive statistics of the study participants at baseline. On average, individuals in the 1-year intervention group were older than those in the group of program dropouts, experienced lower pre-treatment earnings, were more often unemployed at the time of program application and disproportionally more often came from eastern Germany. The 2-year intervention group, on the other hand, was marginally younger than the group of program dropouts, had a relatively higher percentage of females, had higher pre-treatment earnings and were less often unemployed at the time of application. The primary medical diagnosis differed only marginally between comparison groups.

**Table 2:** Baseline Characteristics of the 1-year re-training intervention group, the 2-year re-training intervention group and the group of program dropouts

Baseline characteristics	1-year re-training (*n = *278)	2-year re-training (*n = *1754)	Program dropouts (*n = *367)
Age in years			
Mean (SD)	41.58 (6.82)	37.87 (6.75)	38.11 (6.98)
Gender			
Females	33%	37%	31%
Income 2003			
Mean (SD)	€10,096 (€11,898)	€13,249 (€12,437)	€11,078 (€11,896)
Income 2004			
Mean (SD)	€5849 (€9280)	€6922 (€10,115)	€5227 (€8991)
Employment status			
Unemployed	62%	44%	50%
Residential region			
Eastern Germany	56%	28%	30%
Medical diagnosis			
ICD 5 (mental disorders)	15%	15%	13%
ICD 13 (Musculoskeletal Disorders)	58%	64%	63%
Actual program participation (months)	9.72	21.19	14.10

Additional control variables used in the analysis but not reported in this output are indicator variables for the level of education and the last registered occupation type. For both groups of variables, there were many observations with missing data. Most individuals in the sample for whom education data was available have no tertiary education, but many have completed an apprenticeship. The individuals in the sample for whom data on the industry type was available, were most frequently formerly employed in qualified manual tasks, (semi-) professions as well as simple manual labor and services

*SD* standard deviation, *ICD* international classification of diseases

The mean duration of program participation was additionally surveyed to provide background information on program implementation. Average program duration was 9.72 months among those who successfully completed a 1-year re-training and 21.19 months among those, who successfully completed a 2-year re-training. Program dropouts, on average, participated in the program for 14.10 months. The long program participation time of program dropouts was linked to the fact, that many of the program dropout cases participated in a 2-year re-training.

### Covariate Balance

Table 3 shows that the mean standardized differences in covariates between re-training graduates and program dropouts, after re-weighting of observations, were small for all measured baseline characteristics. The differences can be considered negligible when compared to a cut-off of 0.10 standard deviations. The largest improvement in covariate balance, between the initial sample and the weighted sample, was attained by reducing the difference in the share of persons from former East German states between the 1-year graduates and the dropout group from 0.53 standard deviations before re-weighting of observations, down to 0.01 standard deviations after re-weighting. Similarly, the difference in income earned in the year 2004 between the 2-year graduates and the dropout group was reduced from 0.18 standard deviations before re-weighting of observations, to 0.01 standard deviations after re-weighting. Summarizing, the analysis of standardized differences suggested that the distribution of covariates between comparison groups were balanced after observations were re-weighted by the inverse probability of being treated.

**Table 3:** Standardized differences before and after re-weighting of observations, 1-year re-training intervention group vs. control group and 2-year re-training intervention group vs. control group

Program dropouts	Initial sample		Weighted sample	
	*n = *367		*n = *794	
1 year graduates	*n = *278		*n = *809	
2 year graduates	*n = *1754		*n = *795	
Baseline characteristics	1 vs. 0	2 vs. 0	1 vs. 0	2 vs. 0
Age	0.50	− 0.04	− 0.07	< 0.01
Female	0.04	0.13	0.04	0.02
Income 2003	− 0.08	0.18	− 0.06	0.01
Income 2004	0.07	0.18	0.05	< 0.01
Income 2004 * Age	0.12	0.17	0.04	< 0.01
Income 2003 * Income 2003	− 0.05	0.16	− 0.01	0.01
Unemployed	0.26	− 0.12	− 0.10	< 0.01
Former East Germany	0.53	− 0.05	< 0.01	0.01
ICD 5 (mental disorders)	0.08	0.06	< 0.01	− 0.02
ICD 13 (MSD)	− 0.12	< 0.01	− 0.07	0.03
Neither Abitur nor apprenticeship	− 0.04	− 0.11	− 0.03	< 0.01
No Abitur but apprenticeship	− 0.08	0.13	0.09	0.00
Abitur	− 0.04	− 0.02	0.06	0.01
University degree	− 0.06	− 0.14	0.06	< 0.01
Agriculture	0.08	0.01	0.01	<0.01
Simple manual labor	0.07	0.03	− 0.11	− 0.01
Qualified manual labor	− 0.13	0.01	− 0.10	< 0.01
Technician/engineer	0.07	− 0.03	− 0.01	− 0.04
Simple services	− 0.02	− 0.06	− 0.07	< 0.01
Qualified services	− 0.11	− 0.02	− 0.06	0.01
(Semi-) professions	− 0.12	0.14	0.06	0.02
Simple commercial/administrative	− 0.08	− 0.07	0.04	< 0.01
Qualified commercial/administrative	0.04	0.03	− 0.02	< 0.01

Initial Sample, standardized differences in covariates between comparison groups as recorded; Weighted Sample, standardized differences in covariates between comparison groups after re-weighting of observations by inverse probability of being treated. The term “Abitur” refers to a set of examinations taken in the final year of secondary school in Germany

### Missing outcome data analysis

Before turning to the main results, trends in missing outcome data are summarized in this section. In particular, the observations with missing or interrupted income or employment data (961 dropped observations) were closely examined in relation to the uptake of a pension due to a reduced earnings capacity. The results of our analysis were as follows: (1) Among all program dropouts, there was a higher proportion of incomplete income or employment data compared to program graduates (39.14% vs. 26.30%). (2) Among observations with incomplete income or employment data, there was a substantially higher proportion of cases with a pension uptake due to a reduced earnings capacity (36.54% vs. 5.42%). (3) Among program dropout cases with incomplete income or employment data, the proportion of persons receiving a pension due to a reduced earnings capacity was higher compared to graduates with incomplete data. While every second dropout case with missing data was associated with pension uptake, less than every third case in the group of graduates with incomplete data, was associated with pension uptake (50.00% vs. 32.55%).

The same analysis was also carried out with regards to the uptake of an old-age pension. While the proportion of persons receiving old-age pension was higher in incomplete data cases, total numbers were very small, thus, likely only having a small effect on the estimated relationships. Only 0.77% of all (complete and incomplete) cases received old age pension between 2006 and 2013.

Taken together, the missing data analysis suggests that disproportionally many cases, which were interrupted or incomplete, thus, not considered in our analysis, were cases in which a pension due to a reduced earnings capacity was awarded. There were more dropouts with incomplete data relative to graduates with incomplete data and there was also a higher proportion of awarded earnings incapacity cases in the program dropout group with missing data compared to the graduates with incomplete or missing employment data.

### Average Treatment Effects (ATE)

Table 4 shows that the re-training programs had a significant influence on measured income and employment outcomes compared to the no re-training scenario. The ATE of 1-year re-training on nominal income was equal to €22,839, the ATE of 2-year re-training was equal to €35,620. This was in comparison to the counterfactual scenario of no completed re-training. Without successfully completed re-training, the average mean income over the 8-year observation period was assessed to be equal to €81,961. Including the effects of inflation, the ATE of 1-year re-training on income was reduced to €21,539, while the ATE of 2-year re-training on income was reduced to €32,776. All estimated differences in income were statistically significant at the one percent level.

**Table 4:** Potential outcome means and average treatment effects (ATE), 1-year and 2-year re-training in comparison to no completed re-training

	Potential-outcome mean (SD)	Average treatment effect (95% CI)
Income (in nominal €)		
No re-training (program dropouts)	€81,961 (€3764)	
1 year re-training	€104,801 (€6063)	€22,839 (€8992, €36,687)**
2 year re-training	€117,582 (€1910)	€35,620 (€27,546, €43,695)***
Income (in 2005 €)		
No re-training (program dropouts)	€75,255 (€3424)	
1 year re-training	€96,793 (€5710)	€21,539 (€8549, €34,528)**
2 year re-training	€108,030 (€1769)	€32,776 (€25,471, €40,081)***
Employment (in days)		
No re-training (program dropouts)	1209 (43)	
1 year re-training	1531 (76)	322 (152, 492)***
2 year re-training	1654 (20)	445 (354, 536)***
Social-security benefits (in days)		
No re-training (program dropouts)	677 (23)	
1 year re-training	377 (25)	− 300 (− 366, − 235)***
2 year re-training	589 (7)	− 88 (− 136, − 42)***
Short-term unemployment (in days)		
No re-training (program dropouts)	185 (11)	
1 year re-training	136 (12)	− 50 (− 82, − 17)**
2 year re-training	162 (5)	− 23 (− 47, 1)
Long-term unemployment (in days)		
No re-training (program dropouts)	373 (26)	
1 year re-training	437 (42)	64 (− 33, 160)
2 year re-training	231 (10)	− 142 (− 196, − 88)***
Earnings incapacity pension (percentage)		
No re-training (program dropouts)	10.1 (1.5)	
1 year re-training	9.1 (1.8)	− 1.1 (− 5.6, 3.5)
2 year re-training	4.3 (0.5)	− 5.8 (− 8.9, − 2.7)***

Accumulative results after 8 years. Long-term UE only from 2006 to 2010. Inverse Probability Weighted Regression Adjustment Method was used to estimate potential-outcome means. The potential-outcome means refer to the average of the outcomes, for a specific level of re-training, given that all individuals would have attained this outcome. The average treatment effect measures the difference in these means. Final values were rounded to the nearest whole number (to the nearest tenth for percentage)

***p < 0.001; **p < 0.01; *p < 0.05

An analysis of the number of days with employment showed that the income gains were mainly the result of more days in employment. The potential-outcome means with successfully completed re-training were significantly larger compared to the potential outcome mean with program dropout. Over the 8-year observation period, the average treatment effect of 1-year re-training on the number of days in employment was equal to 322 days, while the ATE of 2-year re-training was equal to 445 days. Both estimates were significant at the one percent level. Additionally, there was a significant decrease in days with social-security and unemployment benefits linked to the completion of both programs. The ATE of 1-year programs on the number of days with social-security benefits was equal to − 300 days, while the ATE of 2-year programs was equal to − 88 days. Both estimates were significant at the one percent level. Moreover, the ATE of re-training on the number of days with short-term unemployment benefits was equal to − 50 days with a 1-year re-training, and − 23 days with a 2-year re-training. The estimated ATE of 1-year re-training on short-term unemployment was significant at the one percent level. The effect of 2-year re-training on the number of days with short-term unemployment benefits was not statistically significant. On the other hand, the mean effect of 1-year re-training on long-term unemployment was not statistically significant, whereas the ATE of 2-year re-training on accumulative days with long-term unemployment benefits was significant at the one percent level. The ATE of 1-year re-training on long term unemployment was equal to 64 days, while the ATE of 2-year programs on the accumulative number of days with long-term unemployment benefits was equal to − 142 days.

The re-training programs also influenced the uptake of a pension due to a reduced earnings capacity. While the likelihood to receive an earnings incapacity pension without completed re-training was equal to 10.1%, it was 9.1% with 1-year re-training and 4.3% with 2-year re-training. The corresponding ATE of 1-year re-training on earnings incapacity pension uptake of − 1.1% was not statistically significant. However, the ATE of 2-year re-training on earnings incapacity pension uptake of − 5.8% was significant at the one percent level.

### Average Treatment Effects on the Treated (ATET)

Table 5 shows that the evaluated re-training programs had a large effect on the graduates’ accumulative incomes and employment days. Without graduation from re-training, 1-year re-training graduates would have earned only €69,939 over the 8-year observation period. With re-training, 1-year program graduates, on average, had earned €24,260 more in comparison. Two-year program graduates, on average, would have earned €84,445 without completed re-training measures, but were able to increase their income as a result of graduating from the re-training programs, on average, by €35,972. The corresponding ATET on real incomes was equal to €22,742 for 1-year program graduates, and €33,097 for 2-year program graduates. All estimated income effects were statistically significant at the one percent level.

**Table 5:** Potential outcome means without re-training among treated individuals and average treatment effects on the treated (ATET), 1-year and 2-year re-training completers in comparison to no re-training scenario

	Potential-outcome mean without completed re-training (SD)	Average treatment effect on the treated (95% CI)
Income (in nominal €)		
1 year re-training	€69,939 (€4601)	€24,260 (€12,805, €35,715)***
2 year re-training	€84,445 (€3935)	€35,972 (€27,743, €44,202)***
Income (in 2005 €)		
1 year re-training	€64,251 (€4250)	€22,742 (€12,150, €33,334)***
2 year re-training	€77,533 (€3637)	€33,097 (€25,495, €40,700)***
Employment (in days)		
1 year re-training	1095 (62)	405 (249, 561)***
2 year re-training	1234 (44)	441 (349, 534)***
Social security benefits (in days)		
1 year re-training	647 (28)	− 316 (− 380, − 251)***
2 year re-training	684 (23)	− 92 (− 140, − 45)***
Short-term unemployment (in days)		
1 year re-training	170 (17)	− 26 (− 66, 13)
2 year re-training	188 (11)	− 24 (− 48, 0)
Long-term unemployment (in days)		
1 year re-training	451 (43)	20 (− 82, 122)
2 year re-training	357 (26)	− 140 (− 194, − 87)***
Earnings incapacity pension (percentage)		
1 year re-training	10.2 (1.9)	− 3.1 (− 7.8, 1.6)
2 year re-training	10.2 (1.5)	− 6.0 (− 9.2, − 2.9)***

Accumulative results after 8 years. Long-term UE only from 2006 to 2010. Inverse Probability Weighted Regression Adjustment Method was used to estimate potential-outcome means. The potential-outcome means of no re-training refer to the average potential outcome, that would have occurred among those that graduated from a specific re-training had they not completed the re-training measures. The average treatment effect on the treated measures the difference in means with and without re-training for the subset of treated individuals. Final values were rounded to the nearest whole number (to the nearest tenth for percentage)

***p < 0.001; **p < 0.01; *p < 0.05

According to our analysis, 1-year program graduates were employed for 405 additional days in comparison to the counter-factual scenario without completed re-training. 2-year program completers were employed for 441 additional days in comparison to the no-training scenario. The estimated effects on employment days were significant at the one percent level. Moreover, 1-year re-training completers had 316 fewer days with social-security benefits compared to the no re-training szenario. Two-year re-training graduates had 92 fewer days on social-security benefits. Both estimated reductions in the number of days with social-security benefits were significant at the one percent level. In addition, 1-year program graduates, on average, had 26 fewer days on short-term unemployment benefits in comparison to the non-training scenario, while 2-year graduates had 24 fewer days. However, differences in days with short-term unemployment benefits were not statistically significant for either group of re-training graduates. Moreover, in comparison to the potential outcome mean without re-training, 1-year program graduates, had 20 additional days on long-term unemployment benefits, whereas 2-year program graduates had 140 fewer days on long-term unemployment benefits. The estimated changes in long-term unemployment benefits were not statistically significant for 1-year program graduates, but significant at the one percent level for 2-year re-training graduates.

Participation in a re-training program also had an effect on the likelihood of an uptake of a pension due to a reduced earnings incapacity. The likelihood of being awarded an earnings incapacity pension was 3.1% lower for completers of 1-year programs, and 6.0% lower for completers of 2-year programs. The baseline likelihood of receiving an earnings incacapacity pension, without successful completion of re-training, was equal to 10.2%. The estimated treatment effect was not statistically significant for 1-year graduates, but significant at the one percent level for 2-year re-training graduates.

### Annual Income Development

**Fig. 2:**
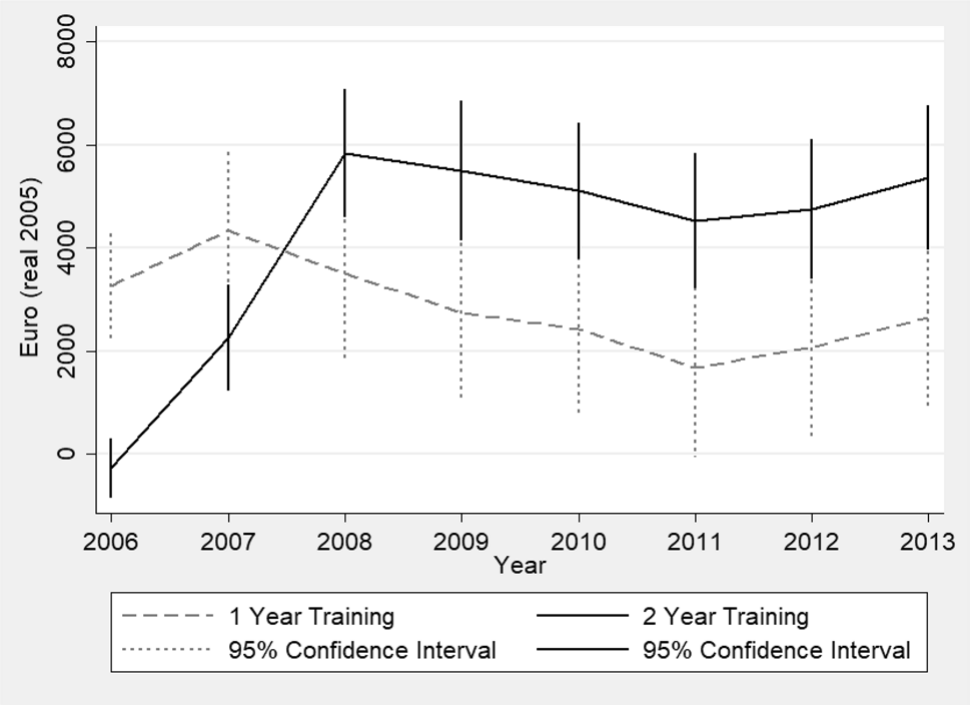
Graphical Analysis of annual real income development 2006–2013 among re-training graduates in comparison to the scenario of unsuccessful completion of re-training; ATET; IPWRA was used to estimate annual treatment effects; vertical lines illustrate 95% confidence interval

Figure 2 illustrates the annual, inflation-adjusted, income development of the re-training graduates in comparison to the scenario without completed re-training. During the years 2007–2013, graduates of both re-training programs had significantly higher income compared to the counterfactual scenario of no-training. The effect of the 2-year intervention was delayed by 1 year due to longer program duration but surpasses the effects of the 1-year programs starting in 2008. The positive effects of vocational re-training on income were sustained until the last year of the observation period. In the year 2013, the (adjusted) difference in real income of 1-year program graduates was €2661, 95% CI [€882, €4439], higher compared to the non-training scenario. Two-year program graduates earned €5369, 95% CI [€3975, €6764], more compared to the counterfactual scenario of no completed re-training.

### Sensitivity Analysis: Only Non-Medical Reasons for Program Dropout

In this last section, the control group was trimmed to program dropouts who reported a non-medical reason for program non-completion. The most prevalent reasons for program non-completion were of a medical nature (48.2%), followed by failed examinations (21.8%) and other performance issues (6.5%) (cf. Table 1). It could be argued that program dropouts who left the program for medical reasons carried some unmeasured confounders linked to their state of health, which negatively affected the employment situation. Thus, it was worth recalculating the model without program dropouts who left the program due to a reported medical reason.

Leaving out data from 177 participants reduced the comparison group to 190 observations (overall n = 2222). The analysis of standardized differences suggested that the distribution of covariates between comparison groups were balanced after observations were re-weighted by the inverse probability of being treated. All differences in covariates were reduced to a negligible level, the necessary condition to carry out the impact analysis. According to the reduced sample analysis, the adjusted differences in income were still significant but slightly reduced in absolute value in comparison to the treatment effects on the full sample. Over the 8-year observation period, the nominal treatment effect on the treated for 1-year program graduates was reduced to €22,451, 95% CI [€8238, €36,664], and for 2-year program graduates to €32,246, 95% CI [€21,568, €42,923]. For the other outcome measures, the estimates were trimmed by a similar margin (~10%).

## Discussion

In this article, we have provided an empirical example of how to use administrative panel data when evaluating re-training programs for people with disabilities. The main research goals of this analysis were linked to finding a viable control group and linking various data sources to examine the long-term effectiveness of vocational re-training measures for a representative group of rehabilitants in Germany. Those effects included changes in individual earnings and days with employment, earnings incapacity pension receipt and changes in the number of days on social-security and unemployment benefits. Based on a sample of 2399 individuals admitted into vocational re-training, we examined the effectiveness of the measures by estimating the average treatment effect (ATE) and the average treatment effect on the treated (ATET) in relation to the case of not having successfully completed re-training.

The treatment effects presented in this study are an important contribution to the empirical knowledge on the influence vocational re-training on the employment re-integration of individuals with disabilities in Germany. This claim is based on the availability of a unique database and the application of appropriate statistical methods, which have allowed a thorough evaluation of the impact of the re-training programs. According to the estimated treatment effects, completing a vocational re-training was associated with significant improvements in the employment status while also reducing dependency on social security benefits. Policies to promote the labor market re-integration of persons with disabilities should consider that vocational re-training may be an effective tool for sustainably improving work participation outcomes.

The choice of method for the analysis of absolute treatment effects in this study adds to the methodological debate on how to estimate program effects from observational data for public policymaking. The main point of debate is whether policymakers can confidently rely on treatment effect estimates obtained from quasi-experimental research settings. Much of the literature indicates that the most common weakness in the quasi-experimental research setting is linked to using an external comparison group. While different training programs exhibit different institutional settings, the foregoing analysis suggests that it is possible, at least for the case of vocational rehabilitation in Germany, to make use of information on program dropouts as an appropriate internal control group to obtain meaningful treatment effect estimates. While this study only included a relatively small number of controls in relation to the number of treated subjects, the analysis of standardized differences has shown that despite the relatively small number of controls, effective re-weighting was carried out, which limited the differences in covariates between comparison groups to a negligible level. This was the prerequisite to carry out the impact analysis.

The results of our impact analysis suggest that both types of vocational re-training programs analyzed significantly improved the income and employment situation of the individuals involved over the first 8 years after program admission. After other factors had been controlled for, graduates of 1-year re-training were on average employed for an additional 405 days, 95% CI [249 days, 561 days], and had earned €24,260 more than without re-training, 95% CI [€12,805, €35,715]. Two-year program completers, on average, were employed for 441 additional days, 95% CI [349 days, 534 days], and had earned €35,972 more than without re-training, 95% CI [€27,743, €44,202]. Moreover, completing a re-training program significantly reduced the number of days on social security benefits and influenced the number of days on unemployment benefits. In addition, the measures also reduced the likelihood of receiving an earnings incapacity pension in comparison to the counterfactual scenario of no re-training. Over the 8-year observation period, one-year program graduates had a 3.1% lower likelihood of being awarded a pension due to a reduced earnings capacity, 95% CI [− 7.8%, 1.6%]; 2-year program graduates had a 6.0% lower likelihood, 95% CI [− 9.2%, − 2.9%].

Whereas the missing data analysis has shown that missing employment data was positively associated with pension uptake, the estimated effects of completing re-training were likely smaller compared to analysis on the full sample. This is because disproportionally many cases, which were interrupted or incomplete, thus, not considered in our analysis, were cases in which a pension due to a reduced earnings capacity was awarded. There were more dropouts with incomplete data relative to graduates with incomplete data and there was also a higher proportion of awarded earnings incapacity cases in the program dropout group with missing data compared to the graduates with incomplete or missing employment data.

Regarding the relative effectiveness of the two interventions studied, the estimated mean employment effects were generally larger for 2-year re-training programs in comparison to 1-year programs (despite longer program duration), indicating that some individuals could have potentially benefitted from allocation into a full, 2-year, re-training program. However, the response to treatment is likely to be heterogeneous and dependent on the individual situation and preferences of the rehabilitants. Program participation depends both on rehabilitant eligibility and on the selection by the person in charge or self-selection by potential participants. In order to better understand the conditions under which allocation into a more involved program could lead to better outcomes, more information on the complex relationship between the applicant and the provider in the application and screening process is needed.

When drawing causal conclusions from the estimated relationships the set-up of the quasi-experiment should always be kept in mind. The association between the duration or level of schooling and earnings does not necessarily imply causality. A potential drawback of this study is that the range of potential variables for calculating the propensity score was limited to a fixed number of registered covariates. Although this study incorporates various proxies to model the earnings ability of the rehabilitants, it was not possible to observe or measure the rehabilitants’ motivation or other soft factors that have an influence on employment outcomes, which may lead to imprecise estimates. Program dropouts participated in the re-training measures before leaving the program, thus, also profiting from the skill training, while at the same time, using up time that could have been spent otherwise if they would have never participated in the measures at all (e.g. to earn money or to get a different training).

On the one hand, failed examination and other performance issues being common among the group of program dropouts’ signal that the former might carry some unmeasured confounders, which could negatively affect their earnings performance. This would mean that the estimated causal relationships in this article were upward biased, due to the negligence of these factors. On the other hand, the argument can be brought forward, that many of the program dropouts likely discontinued their training because they found another job opportunity (perhaps even linked to the benefits received from participating in re-training). This would mean that the treatment effects were underestimated, since some of the wage improvement in the control group might have been due to knowledge and skills attained during the period of rehabilitation. Incorporating proxies for these “factors” into future analysis could contribute to a better understanding of individual education and work reintegration trajectories.

To better understand the individual needs and responses to training, it would also be relevant to further explore the perspective of the individual before and during the period of rehabilitation as well as the role of the provider in programmatic decision making. While about half of program dropouts left the program for medical reasons, further explorative analysis is needed to improve the assistance provided during the period of rehabilitation to prevent the occurrence of medically related dropouts. This includes knowing more about the barriers with regards to the access to the re-training measure, which would allow a more accurate prediction of when re-training is medically appropriate and what type of additional assistance (accommodation) is needed for the measures to have the greatest chance of success.

In addition, further analysis could investigate whether program dropout could have been predicted before the start of the measures based on better screening and program allocation mechanisms. Apart from medical reasons for program dropout, failed examinations and other performance issues were the other main reasons why individuals were not able to finish a re-training program. Consequently, future research should further investigate possible assistance that can be provided during the rehabilitation to increase graduation rates. Moreover, the outcome of allocation practices should be further examined: Were the individuals satisfied with the occupational re-training choice? Would they rather have enrolled in a different trade or occupation? Were there other reasons that influenced how well the rehabilitants performed in the examinations? Given the large benefits associated with completed re-training and the comparatively large costs of these measures, these are relevant questions.

## Footnotes

1 All estimations were carried out using the statistical Software Stata 14.2. StataCorp LLC, 4905 Lakeway Drive, College Station, Texas 77845-4512, USA.

2 Statistical analysis was performed using the group of *“teffects”* commands.
